# Association of Liposome-Encapsulated Trivalent Antimonial with Ascorbic Acid: An Effective and Safe Strategy in the Treatment of Experimental Visceral Leishmaniasis

**DOI:** 10.1371/journal.pone.0104055

**Published:** 2014-08-08

**Authors:** Renata A. O. Castro, Neila M. Silva-Barcellos, Carolina S. A. Licio, Janine B. Souza, Míriam C. Souza-Testasicca, Flávia M. Ferreira, Mauricio A. Batista, Denise Silveira-Lemos, Sandra L. Moura, Frédéric Frézard, Simone A. Rezende

**Affiliations:** 1 Programa de Pós-Graduação em Ciências Farmacêuticas – Cipharma, Universidade Federal de Ouro Preto, Ouro Preto, Minas Gerais, Brasil; Coordenadoria da Área de Ciências Biológicas, Instituto Federal de Minas Gerais - Campus Ouro Preto, Ouro Preto, Minas Gerais, Brasil; Programa de Pós-Graduação em Ciências Biológicas – NUPEB, Universidade Federal de Ouro Preto, Ouro Preto, Minas Gerais, Brasil; Departamento de Fisiologia e Biofísica, Universidade Federal de Minas Gerais, Belo Horizonte, Minas Gerais, Brasil; Technion-Israel Institute of Technology Haifa 32000 Israel, Israel

## Abstract

**Background:**

Visceral leishmaniasis (VL) is a chronic debilitating disease endemic in tropical and subtropical areas, caused by protozoan parasites of the genus *Leishmania*. Annually, it is estimated the occurrence of 0.2 to 0.4 million new cases of the disease worldwide. Considering the lack of an effective vaccine the afflicted population must rely on both, an accurate diagnosis and successful treatment to combat the disease. Here we propose to evaluate the efficacy of trivalent antimonial encapsulated in conventional liposomes, in association with ascorbic acid, by monitoring its toxicity and efficacy in BALB/c mice infected with *Leishmania infantum*.

**Methodology/Principal Findings:**

Infected mice were subjected to single-dose treatments consisting in the administration of either free or liposome-encapsulated trivalent antimony (SbIII), in association or not with ascorbic acid. Parasite burden was assessed in the liver, spleen and bone marrow using the serial limiting dilution technique. After treatment, tissue alterations were examined by histopathology of liver, heart and kidney and confirmed by serum levels of classic biomarkers. The phenotypic profile of splenocytes was also investigated by flow cytometry. Treatment with liposome-encapsulated SbIII significantly reduced the parasite burden in the liver, spleen and bone marrow. Co-administration of ascorbic acid, with either free SbIII or its liposomal form, did not interfere with its leishmanicidal activity and promoted reduced toxicity particularly to the kidney and liver tissues.

**Conclusions/Significance:**

Among the evaluated posological regimens treatment of *L. infantum-*infected mice with liposomal SbIII, in association with ascorbic acid, represented the best alternative as judged by its high leishmanicidal activity and absence of detectable toxic effects. Of particular importance, reduction of parasite burden in the bone marrow attested to the ability of SbIII-carrying liposomes to efficiently reach this body compartment.

## Introduction

Leishmaniasis constitutes a series of neglected tropical diseases, endemic in 98 countries and distributed in at least five continents worldwide [1]. Although under notification of cases has always been a problem for this group of illnesses, it is estimated that 0.2 to 0.4 million cases of visceral leishmaniasis (VL) occur annually [1]. In the Americas, the etiologic agents of VL are represented by *Leishmania infantum* and *Leishmania amazonensis*; these species are transmitted between definitive hosts by insects of the genus *Lutzomyia* [2], [3]. As a result of an intricate life cycle, establishment of the disease in the vertebrate host is achieved through a series of immune evasion mechanisms employed by the parasite [4]–[6]. Once the parasite is internalized by phagocytic cells, proliferation occurs only through the amastigote form, which efficiently develops survival mechanisms to defeat the hostile microenvironment of a phagolysosome [4]. Consequently, a successful anti-*Leishmania* drug must be able to reach the intracellular parasite, wherever the hosting cell might be, including tissues associated with low drug bioavailability such as the bone marrow.

Historically, tartar emetic (SbIII complexes) was the first compound used to treat leishmaniasis [7]. However, the accumulated reports on the occurrence of severe side effects caused its removal from the market [8]. To date, successful treatments (e.g., SbV, amphotericin B, paramomycin or miltefosine) require complex posological regimens which are often associated with pronounced side and toxic effects, posing serious restrictions to their utilization [2], [8], [9]. In order to overcome such limitations, leishmaniasis treatment in Europe and the USA has been consolidated upon the utilization of AmBisome (Gilead Sciences, Inc.), a liposomal formulation of amphotericin B [9]. The development of liposome delivery systems, applied to classical free drugs, has proved successful during treatment of human leishmaniasis [10]. In this context, the reintroduction of SbIII, targeting the major parasite proliferation foci whilst preserving leishmanicidal activity, would probably minimize its cellular and tissue-associated toxicities.

Ascorbic acid, a classical antioxidant molecule, has been shown to alleviate tissue damage during leukemia treatment with arsenic trioxide, possibly by neutralizing the effects of increased oxidative stress [11], [12]. Considering the well known toxic effects of antimonials and the recent observation that co-administration of SbV and ascorbic acid also preserves liver function in the murine model of visceral leishmaniasis [13], here we seek to investigate whether the association of ascorbic acid with free and liposomal SbIII would ameliorate any potential damage in the liver, heart and kidney tissues of mice.

## Methods

### Chemicals

Potassium antimony (III) tartrate hydrate, here referred to as SbIII, and Diestearoylphosphatidylcholine were obtained from Sigma (Sigma-Aldrich, USA). A commercially available sterile solution of ascorbic acid, at 100 mg/mL, was obtained from Hypofarma, Brazil. Cholesterol was obtained from Lipoid GmBh, Germany.

### Experimental Animals and Ethics Statement

Throughout this study isogenic BALB/c mice, aged between 6 to 8 weeks, were maintained in the ‘Centro de Ciência Animal da Universidade Federal de Ouro Preto (CCA/UFOP)’ in individual plastic cages containing four animals per group. These were kept at temperatures ranging from 21 to 25°C, under a 12 hour's dark and light cycle. Animals were given water and a commercial rodent diet (Nuvilab CR-II, Brasil) *ad libitum*. The adopted procedures in this study were in accordance with the National Council on Animal Experiments and Control (CONCEA-MCT-Brazil) guidelines and approved by the ‘Comitê de Ética no Uso de Animais da UFOP (CEUA/UFOP)’ under protocol numbers 135/2011 and 25/2011.

### Infection

*L. infantum* (C43 strain) used in this study, gently provided by Dr. Alexandre Barbosa Reis (Laboratório de Pesquisas Clínicas - Cipharma / UFOP), was firstly isolated from a symptomatic dog and subsequently typed by Random Amplification of Polymorphic DNA. Parasites were cultivated at 25 ± 1°C in Grace's Insect Medium pH 6.5 (Sigma, USA) supplemented with 10% heat-inactivated fetal calf serum (SFB – LGC, Brasil), 2 mM L-glutamine and 100 U/mL G penicillin. When the parasite culture reached the stationary phase of growth, promastigotes were collected, washed in sterile PBS (pH 7.2) and 1x10^7^ cells inoculated per female BALB/c mice, by intravenous administration through the tail vein. *L. infantum* infected BALB/c mice (n = 4) were maintained in the bioterium for six weeks prior to administration of the different treatment regimens.

### Liposome preparation

Unilamellar liposomes at 120 g/L were prepared with diestearoylphosphatidylcholine (Sigma, USA) and cholesterol (Lipoid GmBh, Germany) at a 5∶3 ratio, respectively, through the freeze and thaw technique, followed by extrusion using a 0.2 µm polycarbonate membrane [14], [15]. These were then dialyzed against PBS pH 7.2 in a cellulose membrane bag over 24 hours. Inductively coupled plasma optical emission spectrometry (ICP-OES, Spectro Ciros CCD), available at the ‘Laboratório de Geoquímica - Departamento de Geologia / DEGEO / UFOP’, was used to determine the SbIII encapsulation efficiency. Mean size and polydispersion index of the liposomal preparations were measured through photon correlation spectroscopy using a Nanosizer N5 (Beckman Coulter, USA) as per the manufacturer's instructions.

### Treatment regimens

Evaluation of the leishmanicidal activity of free or liposome-encapsulated SbIII co-administered or not with ascorbic acid required the following experimental groups: 1) PBS; 2) free trivalent antimony (SbIII) at 9 mg Sb/kg; 3) empty liposomes; 4) SbIII encapsulated liposomes at 9 mg Sb/kg; 5) ascorbic acid at 300 mg/kg; 6) association of free SbIII (9 mg Sb/kg) plus ascorbic acid (300 mg/kg) and 7) association of SbIII encapsulated liposomes (9 mg Sb/kg) plus ascorbic acid (300 mg/kg). Each treatment regimen was administered to *L. infantum* infected mice (n = 4, per experimental group) as a single dose through the intraperitoneal route.

### Parasite quantification

*L. infantum*-infected mice were euthanized using a combination of Ketamine (Syntec, Brasil) at 24 mg/kg plus Xylazine (Sespo, Brasil) at 12 mg/kg and the parasite load associated to the liver, spleen and bone marrow determined by the limiting dilution technique [16], with minor modifications [17]. Briefly, each animal was submerged in 70% ethanol for 2 min, and under aseptic conditions, a liver fragment, the spleen and the left leg were removed. Both the remaining liver, its fragment and the spleen were weighted and further maintained in ice-cold DMEM medium (Sigma, USA) pH 7.2 supplemented with 1% fetal bovine serum, 2 mM L-glutamine and 100 U/mL G penicillin. The liver fragment and the spleen of each animal were individually macerated in DMEM, using a 2 mL potter device (Corning, USA). The obtained supernatant was collected and placed in 15 mL centrifuge tubes. The whole supernatant obtained by maceration of the liver fragment and 1/5 of the volume obtained by maceration of the spleen were submitted to centrifugation at 42 x *g* for 1min at 4°C for the removal of any tissue debris. Parasite load in the bone marrow was evaluated by first dissecting the left leg for recovery of the femur and tibia and, after cutting out their epiphyses, approximately 5 mL of supplemented DMEM was injected until complete removal of the cellular content. All biological material obtained with the processing of liver, spleen and bone marrow was centrifuged at 1540 x *g* for 10 min at 4°C and the cell pellet suspended in 500 µL of supplemented Grace's Insect Medium pH 6.5. The obtained cell suspensions were distributed in duplicates using sterile 96 flat-well plate, followed by 1∶5 serial dilutions until filling up the entire plate, using the same medium. Parasites were quantified after 7 days in culture at 25°C, by registering the reciprocal of the highest dilution positive for motile parasites. This number was considered to be the concentration of parasites per milligram of tissue. Multiplication of this concentration by the whole-organ weight provided the total organ parasite burden.

### Histopathology and Biochemical Analyses

For histopathological analysis, the remaining liver tissue, the kidneys and heart of each mouse were fixed in 10% buffered formaldehyde pH 7.4 for at least 48h. Fragments measuring approximately 1 cm^2^ were obtained from the organs and automatically processed for histological analysis using a LUPETEC- PT05 equipment. Tissue fragments were included in 60% paraffin, after which 5 µm sections were obtained using a microtome, followed by hematoxylin-eosin staining. Images were digitalized at the NUPEB's Multiuser Facility using a Leica/ DM5000 optical microscope through the *Leica Application Suite* software (version 2.4.1). For morphometric analyses of liver sections, the ratio obtained by dividing the total granulomatous area by the total tissue area was determined (n = 3 / per experimental group). Analyses were performed in a single slide from each of the three animals investigated. Sections were viewed with a 40× objective and images were digitalized by Leica DFC340FX microcamera associated with Leica DM5000B microscopy. All images were analyzed using the image processing and analysis software Leica Qwin V3.

Blood of all animals under experimentation was withdrawn by cardiac puncture and the serum obtained by centrifugation at 164 x g for 15 min at room temperature. Each serum was processed by the fully automatic random access clinical analyzer Metrolab 2300 (Wiener), prior to loading the respective calibrator and specific controls for Bioclin-Quibasa diagnostic kits (Calibrator – Biocal K072; Normal control - Biocontrol NK073 and Pathologic control - Biocontrol PK074). All used reagents were kindly provided by the Bioclin-Quibasa Company through its ‘*Bioclin at the Scientific Laboratory*’ initiative. Kidney's performance was accessed through measurement of creatinine levels. Hepatic function was evaluated through serum activities of aspartate (AST) and alanine aminotransferases (ALT), whereas for cardiac monitoring, levels of AST and creatine kinase MB isoenzyme (CK-MB) were obtained.

### *Ex-vivo* immunophenotyping of splenocytes

From the remaining 4/5 cell suspension obtained with the maceration of the spleen, 1×10^6^ splenocytes were counted and added 0.5 µg of the homemade IgG anti-Fc receptors CD16 and CD32. After 5 min incubation at room temperature, splenocytes were transferred to polystyrene tubes in the presence of diluted anti-mouse cell surface mAbs for 30 min, at room temperature in the dark. The following mAbs were used: anti-CD3 (PE, Clone - 145–2C11, Bio Legend); anti-CD4 (FITC, Clone - RM4-5, Bioscience); anti-CD8 (PerCP-Cy5.5, Clone - 53–6.7, Bioscience); anti-CD19 (FITC, Invitrogen); anti-F4/80 (FITC, Clone - BM8, Bio Legend); anti-MHCII (PE-Cy 5, Clone - M5/114.15.2, Bioscience) and anti-CD86 (PE, Clone - GL1, Bioscience). After incubation, the cells were washed in PBS pH 7.2 and recovered by centrifugation at 210 x *g* for 10 min at 4°C. Cells were washed once again in PBS pH 7.2 and immediately fixed by addition of 200 µL of FACS fix solution (10 g/L paraformaldehyde, 1% sodium cacodylate, 6.67 g/L sodium chloride, pH 7.2). Data collection was performed using a flow cytometer FACScalibur (Becton Dickinson, USA). The FlowJo software was used for data analysis (Tree Star, USA).

### Statistical analyses

Statistical analyses were conducted using GraphPad Prism 5. Each set of results was firstly checked for normal distribution using Kolmogorov-Smirnov, D'Agostinho & Pearson and Shapiro-Walk tests. Normally distributed data were analyzed through one-way-ANOVA followed by Tukey’s post hoc test. For non-normally distributed data a Kruskal–Wallis test followed by Dunn’s post hoc tests were used. Statistically significant differences were assumed when *p* <0.05.

## Results

### The *L. infantum* (C43 strain) colonizes liver, spleen and efficiently reach the bone marrow in the murine model

**Figure 1:**
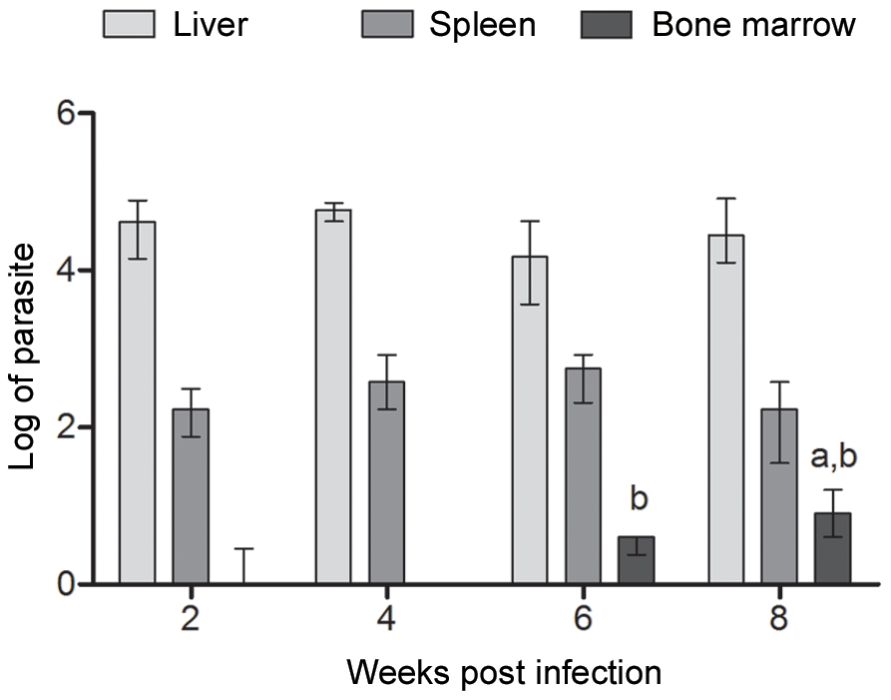
Parasite burden associated to the liver, spleen and bone marrow from *L. infantum* infected mice. Parasite burden is expressed as log_10_ (number of parasites) +1 for each individual organ (n = 4, per experimental group) and represented as median ± interquartile range of two independent experiments. Statistically significant differences were assumed when p<0,05 in relation to (a) two weeks post-infection and (b) four weeks post-infection.

Before quantification of the leishmanicidal activity of liposome-encapsulated SbIII, we first sought to determine the parasite burden associated to the liver, spleen and bone marrow during the 2^nd^ to 8^th^ week's post-infection interval. The limiting dilution technique revealed the presence of the parasite at significant levels in the liver and spleen at the 2^nd^ week post-infection (Figure 1). Parasite levels associated with these organs did not vary significantly for mice euthanized at the 4^th^, 6^th^ and 8^th^ weeks post-infection. On average, parasite burden in the liver was approximately 50% higher than that observed for the spleen. Comparatively, very low levels of parasites were detected in the bone marrow at the 2^nd^ and 4^th^ weeks post-infection. In contrast, at the 6^th^ week post-infection parasite levels in the bone marrow reached 16% of that found in the liver increasing to approximately 20% in animals euthanized at the 8^th^ week post infection. At this point, parasite levels in the bone marrow reached 42% of those found in the spleen.

### Conventional unilamellar liposomes are an efficient drug-delivery system of SbIII for the treatment of murine visceral leishmaniasis

**Figure 2:**
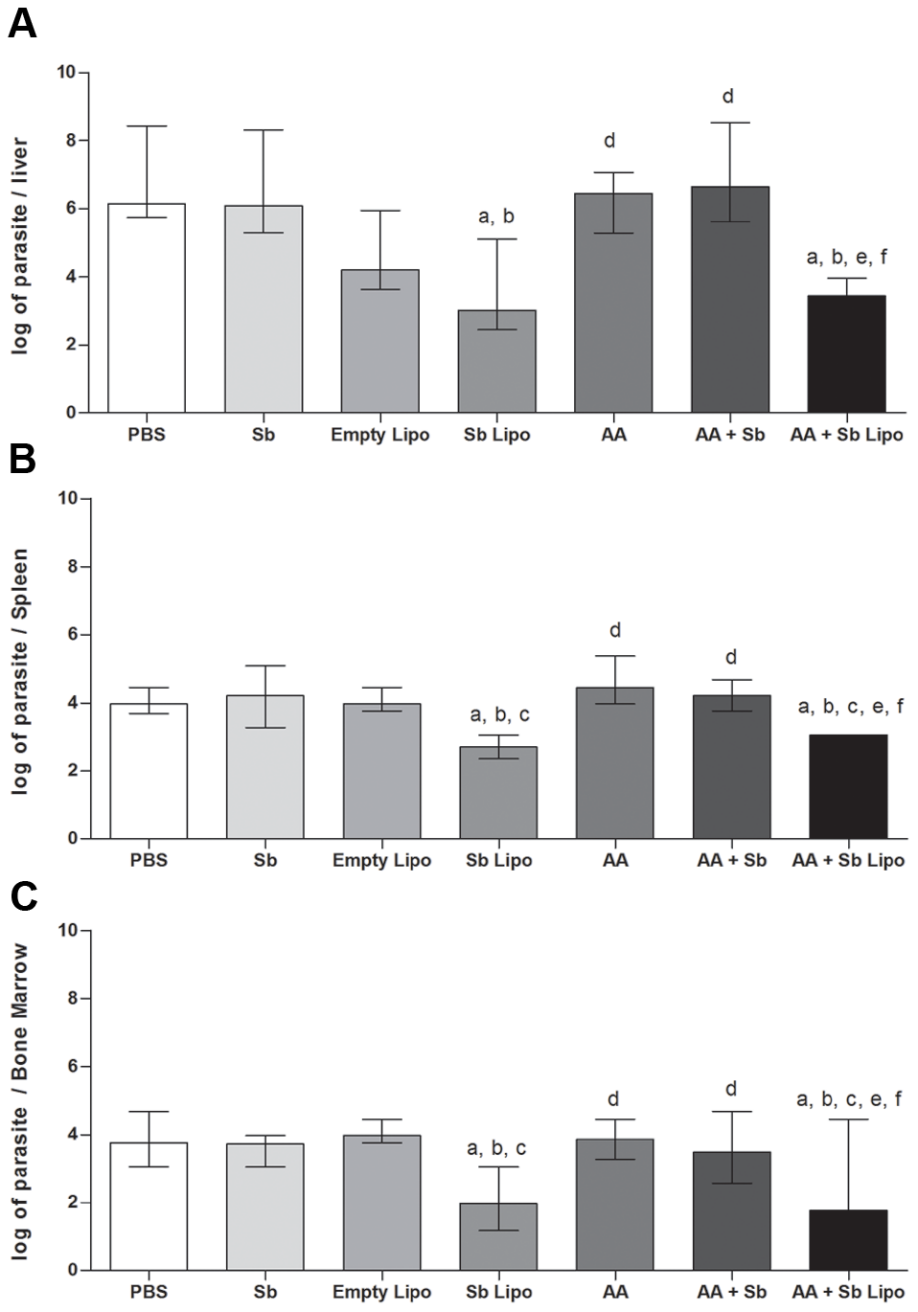
Parasite burden associated to different organs from *L. infantum* infected mice submitted to treatment regimens. (A) Liver, (B) Spleen and (C) Bone marrow. (PBS) phosphate-buffered saline, (Sb) trivalent antimony, (Empty Lipo) empty liposomes, (Sb Lipo) antimony entrapped liposomes, (AA) ascorbic acid, (AA+Sb) ascorbic acid in association with antimony, (AA+Sb Lipo) ascorbic acid in association with antimony entrapped liposomes. Parasite burden is expressed as log_10_ (number of parasites) +1 for each individual organ (n = 4, per experimental group) and represented as median ± interquartile range of two independent experiments. Statistically significant differences were assumed when p<0,05 in relation to (a) PBS; (b) Sb; (c) Empty Lipo; (d) Sb Lipo; (e) AA and (f) AA+Sb.

On the 14^th^ day following the administration of the different treatment regimens, parasite quantification in the liver, spleen and bone marrow revealed reduction in parasitism only for animals treated with SbIII entrapped in liposomes. Characterization of such liposomes showed median size of 222.5 nm, 0.214 polydispersion index and 15% encapsulation efficiency for a preparation containing 4 mg SbIII/mL. Administration of the SbIII liposomal formulation, as a single dose, resulted in 47%, 33% and 47% reduction in parasite burden associated with the liver, spleen and bone marrow, respectively (Figure 2). Parallel administration of ascorbic acid with either free SbIII or SbIII entrapped in liposomes did not alter the parasite burden for the investigated organs. At the employed dose, reductions in parasite levels following treatment with free SbIII were not observed.

### Liposomal SbIII in association with ascorbic acid alleviates the major tissue alterations promoted by antimonials

**Figure 3:**
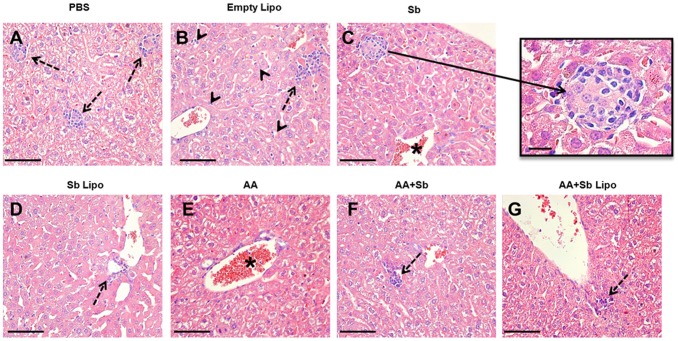
Histopathological analyses of the liver from *L. infantum* infected mice submitted to different treatment regimens. (A) Phosphate-buffered saline (PBS). (B) Empty liposomes (Empty Lipo). (C) Trivalent antimony (Sb). (D) Antimony entrapped liposomes (Sb Lipo). (E) Ascorbic acid (AA). (F) Ascorbic acid in association with antimony (AA+Sb). (G) Ascorbic acid in association with antimony entrapped liposomes (AA+Sb Lipo). Bar  = 50 µm. Insert  = 25 µm, representing an immature granuloma. Dashed arrow  =  Inflammatory cell foci. Arrowhead  =  Kupffer cells exhibiting hypertrophy. * Hyperemic vessels.

**Figure 4:**
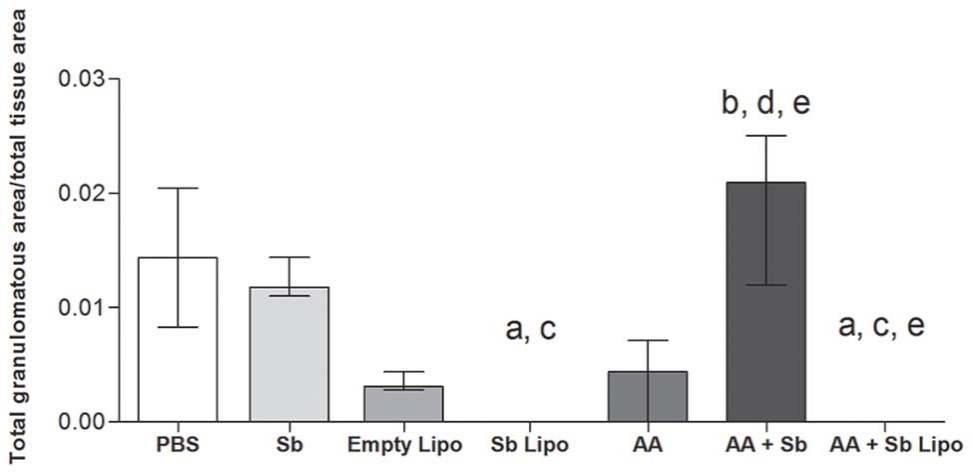
Quantification of granulomatous tissue in the liver of *L. infantum* infected mice submitted to treatment regimens. (PBS) phosphate-buffered saline, (Sb) trivalent antimony, (Empty Lipo) empty liposomes, (Sb Lipo) antimony entrapped liposomes, (AA) ascorbic acid, (AA+Sb) ascorbic acid in association with antimony, (AA+Sb Lipo) ascorbic acid in association with antimony entrapped liposomes. Y axis represents the ratio obtained by dividing the total area occupied by granulomatous tissue (µm^2^) by the total area of liver tissue (µm^2^) for each individual slide (n = 3, per experimental group). Results are represented as median ± interquartile range. Statistically significant differences were assumed when p<0,05 in relation to (a) PBS; (b) Sb; (c) Empty Lipo; (d) Sb Lipo; (e) AA; (f) AA+Sb.

Histopathological analyses of the hepatic tissue demonstrated that infected animals treated with PBS, empty liposomes, free SbIII, AA and AA + Sb exhibited a typical granulomatous inflammation (Figures 3A–C). Quantitative analyses of the tissue area occupied by granulomatous inflammation, in animals belonging to the aforementioned groups, statistically confirmed such finding (Figure 4). In particular, the cell exudate was mainly composed of macrophages and epithelioid cells organized in immature granulomas (Figure 3C, insert). Intralobular focal infiltrates of lymphoplasmocytes were also observed (Figures 3A and 3B, dashed arrows). A diffuse increase in the number of Kupffer cells within the sinusoidal spaces was noted, with the cells exhibiting hypertrophy (Figure 3B, arrowhead). Some centrolobular hepatocytes displayed a moderate degree of hydropic degeneration. The centrolobular veins and the hepatic vein branches, at the portal spaces, were highly hyperemic containing a moderate mononuclear infiltrate as a perivasculitis (Figure 3C, 3E, asterisked areas). For animals treated with liposomal SbIII in association or not with ascorbic acid, hepatic granulomas were not observed. More effectively, the combination of liposomal SbIII with ascorbic acid resulted in pronounced reductions in the inflammatory infiltrate and associated hyperemia when compared to treatment with liposomal SbIII only. Actually, animals treated with the liposomal SbIII/ascorbic acid combination exhibited, at the most, a discrete hyperemia with no alterations on the morphology of Kupffer cells at the sinusoidal spaces (Figure 3G).

**Figure 5:**
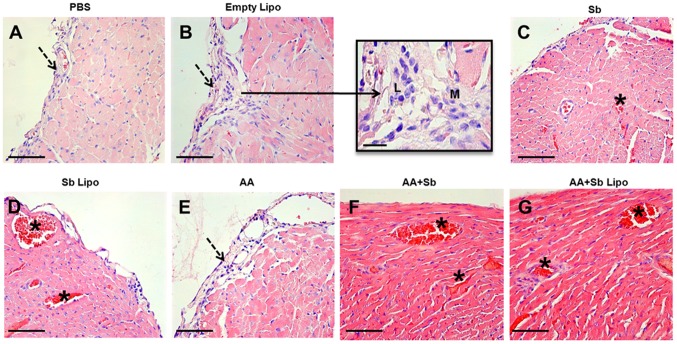
Histopathological analyses of the heart from *L. infantum* infected mice submitted to different treatment regimens. (A) Phosphate-buffered saline (PBS). (B) Empty liposomes (Empty Lipo). (C) Trivalent antimony (Sb). (D) Antimony entrapped liposomes (Sb Lipo). (E) Ascorbic acid (AA). (F) Ascorbic acid in association with antimony (AA+Sb). (G) Ascorbic acid in association with antimony entrapped liposomes (AA+Sb Lipo). Bar  = 50 µm. Insert  = 25 µm, representing an inflammatory infiltrate of lymphocytes (L) and macrophages (M). Dashed arrow  =  subendocardial inflammatory infiltrate composed of mononuclear cells. * Active hyperemia.

Analysis of the cardiac tissue revealed no architectural or cellular degeneration for any of the treatment groups. For infected animals treated with PBS, empty liposomes and ascorbic acid, a discrete and diffuse subendocardial inflammatory infiltrate, composed of mononuclear cells, was observed (Figure 5A, 5B and 5E, respectively). These tissue alterations were not detected for infected animals treated with free SbIII, liposome-entrapped SbIII, and the same treatments associated with ascorbic acid (Figure 5C, 5D, 5F and 5G). Notably, administration of SbIII, irrespective of its formulation, promotes an active hyperemia throughout the myocardium which was not reversed by co-administration of ascorbic acid (Figure 5C, 5D, 5F and 5G, asterisked areas).

**Figure 6:**
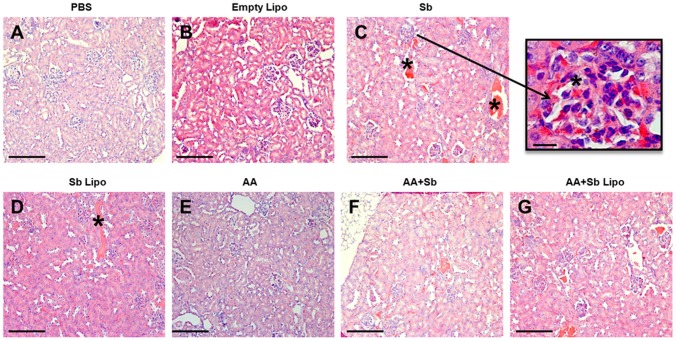
Histopathological analyses of the kidney from *L. infantum* infected mice submitted to different treatment regimens. (A) Phosphate-buffered saline (PBS). (B) Empty liposomes (Empty Lipo). (C) Trivalent antimony (Sb). (D) Antimony entrapped liposomes (Sb Lipo). (E) Ascorbic acid (AA). (F) Ascorbic acid in association with antimony (AA+Sb). (G) Ascorbic acid in association with antimony entrapped liposomes (AA+Sb Lipo). Bar  = 50 µm. Insert  = 25 µm, representing glomerular hyperemia. (A), (B) and (E) renal parenchyma exhibiting normal aspect. (C), (D), (F) and (G) renal parenchyma exhibiting active hyperemia. * Hyperemic vessels.

In the kidney tissue, no alterations in the morphology of glomerular mesangial cells, glomerulonephritis or the presence of hyaline intratubular cylinders were observed in any of the treated groups. The hyperemia found in the cardiac tissue was also notable in the kidney for animals undergoing SbIII treatment (Figure 6C, 6D, 6F and 6G, asterisked areas). Nevertheless, in the kidney hyperemia varied from discrete to moderate. Remarkably, treatment with liposome-entrapped SbIII promoted a reduction in the glomerular congestion (Figure 6D), which was also much alleviated when such regimen was applied in association with ascorbic acid (Figure 6G).

Although some degree of inflammation was detected for the three investigated organs, this did not prove sufficient to alter significantly four classic serum biomarkers of tissue injury: hepatic transaminases (AST and ALT), CK-MB and creatinine (Figure S1). In addition, quantitative evaluation of splenocytes revealed no significant differences in the number of cell populations represented by T (CD4/CD8) and B lymphocytes (Figures S2 and S3, respectively). Finally, the number of spleen macrophages as well as the expression of CD86 and MHCII, two known markers of cell activation, did not vary among the different treatment groups (Figure S4).

## Discussion

In this investigation, we first aimed to determine the time course and level of parasitism caused by *L.infantum* (C43 strain) in the murine model. Such information was of paramount importance to establish the timing of therapeutic intervention and define the experiment's endpoint for data collection. Comparative analyses of parasite burden in the liver and spleen demonstrated early and high levels of parasitism detectable at 2 weeks post-infection; these remained fairly constant in those organs up to week 8. Our data differs from that described for *Leishmania chagasi* (MHOM/BR/1974/M2682 strain) which peaks at four weeks post-infection in the liver of infected mice [18]. We therefore selected week 6 for administration of the different treatment regimens and two weeks afterwards for data collection. This also guaranteed accurate evaluation of bone marrow parasitism at 8 weeks post-infection using the limiting dilution technique.

Prior to administration of the different treatment regimens, characterization of our proposed liposomal preparation revealed an average SbIII encapsulation efficiency of 15%. For SbV-entrapped liposomes encapsulation efficiency was reported to vary from 8 to 50%, depending on the method of preparation [19]. Extrusion of multilamellar liposomes produced a preparation of unilamellar liposomes displaying a polydispersion index < 0.3, consistent with monodisperse particles [20]. Particle homogeneity is of relevance as it may diminish bias related to tissue bioavailability of the formulation. We selected the intraperitoneal route for administration as it provides high drug concentration in the bloodstream for a sustained period [21]. When liposomes are administered through this route, lymphatic absorption is known to occur regardless on the particle size [22].

The recommended dose for leishmaniasis treatment using non-vectored SbV is 20 mg Sb/kg/day [23]. Supported by ongoing toxicological investigations of liposomal SbIII from our group (unpublished results) we established that such formulation, administered at 9 mg/kg, could represent a safe and effective dose. In fact, following treatment, only animals submitted to liposomal SbIII exhibited reduction in parasite burden for all investigated organs. Such findings confirmed the ability of liposomes to target the active compound into tissues containing cells of the mononuclear phagocyte system [24]. In our study, this was demonstrated by the significant reduction in parasite burden associated with the liver and spleen, using SbIII-containing liposomes. The reduction of parasitism at the bone marrow was also of particular importance. Given that the latter is another site for resident phagocytic cells, the 200 nm particles generated proved of suitable size to deliver active SbIII to this compartment. This is in agreement with the reported preferential accessibility of the bone marrow for liposomes ≤ 200 nm diameter [25]. It has been postulated that the smaller the liposome particle, the longer its circulation time in the bloodstream [23]. A sustained period in the circulation would permit a higher number of particles reaching the bone marrow as a way to overcoming their rapid uptake and processing by phagocytes in the liver and spleen [26]. Schettini et al, 2006 demonstrated that SbV-entrapped liposomes, exhibiting an average size of 410 nm promoted a three-fold increase in the concentration of SbV in the bone marrow compared to 1200 nm carrier particles [25].

Reduction in parasite burden was also obtained when SbV-containing liposomes were administered to infected dogs in comparison to treatment with free SbV [25], [27]. Thus, the efficacy of the liposomal delivery system relies on its subsequent capture by cells of the mononuclear phagocyte system, irrespective of which antimonial is employed [28]. This process can be facilitated by opsonization of liposomes as they encounter blood constituents [29]. After internalization by phagocytosis, liposomes are degraded by lysosomal phospholipases and active Sb, now inside phagolysosomes, can either be excreted or diffuse through the cytosol [30]. In the latter case, it is hypothesized that Sb promotes amastigote death by interfering with diverse cellular processes such as the formation of stable thiol complexes [31], [32], inhibition of trypanothione reductase (TR) [33], [34] and binding to zinc finger proteins [35], leading to irreversible cell damage.

Based on the reported efficacy and reduced toxicity of trivalent arsenic co-administered with ascorbic acid during treatment of leukemia patients [36], [37], it was wondered whether tissue damage promoted by SbIII could be alleviated using a combined therapy for the treatment of leishmaniasis. Therefore, it was assumed that SbIII in association with ascorbic acid would promote reduced tissue toxicity, whilst preserving its leishmanicidal activity. This assumption relied on the commonalities observed when the mechanistic toxicology of arsenic was compared to that of antimony [38]. In agreement with such conjecture, inclusion of ascorbic acid in the different treatment regimens did not interfere with SbIII's ability to promote parasite elimination in the liver, spleen and bone marrow. Given that SbIII can decrease the buffering capacity of intracellular thiols, with consequent reduction in the levels of trypanothione, glutathione and parallel inhibition of TR [34], co-administration of ascorbic acid appeared to ameliorate the cellular stress promoted by increased reactive oxygen species. This might indicate a potential mechanism to prevent exacerbated tissue damage. Although it has been shown that *Leishmania* parasites can potentially synthesize ascorbic acid, a number of questions remains to be answered to fully elucidate its particular role in parasite survival/viability [39]. It is otherwise possible that the parasite may somehow benefit from the externally added ascorbic acid but the accumulated evidence points to a dominant leishmanicidal effect caused by antimonials, when these two are co-administered.

In fact, histopathological analyses of the liver from infected animals given the combined liposomal SbIII plus ascorbic acid therapy revealed a pronounced reduction in the associated inflammatory infiltrate. Overall, the hepatic tissue resembled that from an uninfected animal, as judged by the absence of granulomas and of alterations on the morphology of Kupffer cells. In agreement with our findings a recent study revealed that association of the pentavalent antimonial Glucantime (Sanofi-Aventis) with ascorbic acid resulted in protection of the mouse liver tissue [13]. Here we have extended the analysis of the beneficial association of SbIII and ascorbic acid to the heart and kidney tissues. Of particular relevance to the kidney tissue, the combined therapy resulted in decreased glomerular congestion, in contrast to the observed light to moderate hyperemia associated with the other treatment regimens. Whether such an effect could prevent alterations in the endothelial system and/or nitric oxide production remains to be determined. Although hepatic toxicity has long been reported for patients under antimonial treatment [40], [41], the literature is scarce on the potential side effects promoted by this compound in the kidney. Moreover, the infection per se can promote kidney impairment, as it has been demonstrated that anti-parasite specific antibodies could lead to renal deposition of immune complexes, a prior step to development of glomerulonephritis in the mouse model [42]. In this scenario, taking into account that renal excretion constitutes the primary route for elimination of antimonials [43], our data provide relevant information on how effects on kidney integrity could be minimized during leishmaniasis treatment.

To our knowledge, the only attempt to treat murine visceral leishmaniasis using SbIII entrapped in multilamellar liposomes was 36 years ago [44]. On that occasion, the authors reported parasite elimination from the liver two days post-infection, following intravenous administration of either one or three doses of liposomal SbIII (20 mg Sb/kg), to *L. donovani* infected animals. In that study, the level of liver infection was evaluated through amastigote counting on impression smears using light microscopy. Here we employed a single intraperitoneal administration of unilamellar liposomes at a much reduced dose. Evaluation of the therapeutic's efficacy occurred only 14 days after treatment, allowing for precise detection of live parasites in infected tissues. In agreement with the former study, it was demonstrated that our SbIII liposomal preparation can promote significant reductions in the number of viable parasites not only associated with the liver and spleen, but also to that disseminated to the bone marrow. Concerning the latter tissue, our data is of particular relevance as failure to reach an effective concentration of antimonials *in situ* has been considered a major cause of unsuccessful treatment and relapse [45].

Finally, our work augmented the proposed benefits of unilamellar liposomes carrying SbIII by demonstrating their reduced toxicity, particularly in combination with ascorbic acid to the liver and kidney tissues. Although the wide utilization of pentavalent antimonials has long been justified by the occurrence of comparatively fewer toxic effects promoted by SbV, a recent study using an accurate and sensitive analytical technique revealed non-negligible levels of SbIII complexes (>30%) as constituents of Glucantime [46]. We believe our investigation will be useful for the proposal of novel therapeutic regimens to treat VL successfully, with the aim of guaranteeing patient compliance whilst minimizing potential side effects.

## Supporting Information

Figure S1 **Monitoring liver, kidney and heart functions for** ***L. infantum*** **infected mice submitted to treatment regimens.** (PBS) phosphate-buffered saline, (Sb) trivalent antimony, (Empty Lipo) empty liposomes, (Sb Lipo) antimony entrapped liposomes, (AA) ascorbic acid, (AA+Sb) ascorbic acid in association with antimony, (AA+Sb Lipo) ascorbic acid in association with antimony entrapped liposomes. (A) Serum activity of aspartate aminotransferase (AST). (B) Serum activity of alanine aminotransferase (ALT). (C) Serum activity of creatine kinase MB isoenzyme (CK-MB). (D) Serum creatinine concentration in mg/mL. Enzyme activity is expressed as U/L. Results are represented as median ± interquartile range of two independent experiments. No statistically significant differences were observed.
(TIF)

Figure S2 ***Ex vivo*** **immunophenotyping of T lymphocytes from** ***L. infantum*** **infected mice submitted to treatment regimens.** (PBS: A-C) phosphate-buffered, (Sb: D) saline trivalent antimony, (Empty Lipo: E) empty liposomes, (Sb Lipo: F) antimony entrapped liposomes, (AA: G) ascorbic acid, (AA+Sb: H) ascorbic acid in association with antimony, (AA+Sb Lipo: I) ascorbic acid in association with antimony entrapped liposomes. (A) Size and granularity profiles of lymphocytes. (B) Granularity profile of CD3^+^ cells. (C-I) Profile of T CD8^+^ and CD4^+^ lymphocytes. (J) Percentage of T (CD4^+^) lymphocytes. (K) Percentage of T (CD8^+^) lymphocytes. In (J) results are represented as mean ± SD whereas for (K) as median ± interquartile range of two independent experiments. No statistically significant differences were observed.
(PDF)

Figure S3 ***Ex vivo*** **immunophenotyping of B lymphocytes from** ***L. infantum*** **infected mice submitted to treatment regimens.** (PBS: A-B) phosphate-buffered, (Sb: C) saline trivalent antimony, (Empty Lipo: D) empty liposomes, (Sb Lipo: E) antimony entrapped liposomes, (AA: F) ascorbic acid, (AA+Sb: G) ascorbic acid in association with antimony, (AA+Sb Lipo: H) ascorbic acid in association with antimony entrapped liposomes. (A) Size and granularity profiles of lymphocytes. (B-H) Granularity profile of CD19^+^ cells. (I) Percentage of B (CD19^+^) lymphocytes. In (I) results are represented as mean ± SD of two independent experiments. No statistically significant differences were observed.
(PDF)

Figure S4 ***Ex vivo*** **immunophenotyping of macrophages from** ***L. infantum*** **infected mice submitted to treatment regimens.** (PBS: A-D) phosphate-buffered, (Sb: E-G) saline trivalent antimony, (Empty Lipo: H-J) empty liposomes, (Sb Lipo: K-M) antimony entrapped liposomes, (AA: N-P) ascorbic acid, (AA+Sb: Q-S) ascorbic acid in association with antimony, (AA+Sb Lipo: T-V) ascorbic acid in association with antimony entrapped liposomes. (A) Size and granularity profiles of splenocytes. (B; E; H; K; N; Q; T) Percentage of F4/80^+^ cells. (C; F; I; L; O; R; U) Percentage of F4/80^+^ CD86^+^ cells. (D; G; J; M; P; S; V) Percentage of F4/80^+^ MHCII^+^ cells. (W) Percentage of F4/80 labeled macrophages. (X) Mean fluorescence intensities for F4/80 and CD86 labeled macrophages. (Y) Mean fluorescence intensities for F4/80 and MHCII labeled macrophages. In (W) and (Y) results are represented as mean ± SD whereas for (X) as median ± interquartile range of two independent experiments. No statistically significant differences were observed.
(PDF)
