# Supplementary figures and images for: Association of Liposome-Encapsulated Trivalent Antimonial with Ascorbic Acid: An Effective and Safe Strategy in the Treatment of Experimental Visceral Leishmaniasis
Source: PLoS One. 2014 Aug 8;9(8):e104055. doi: 10.1371/journal.pone.0104055 (PMC4126701; doi:10.1371/journal.pone.0104055)

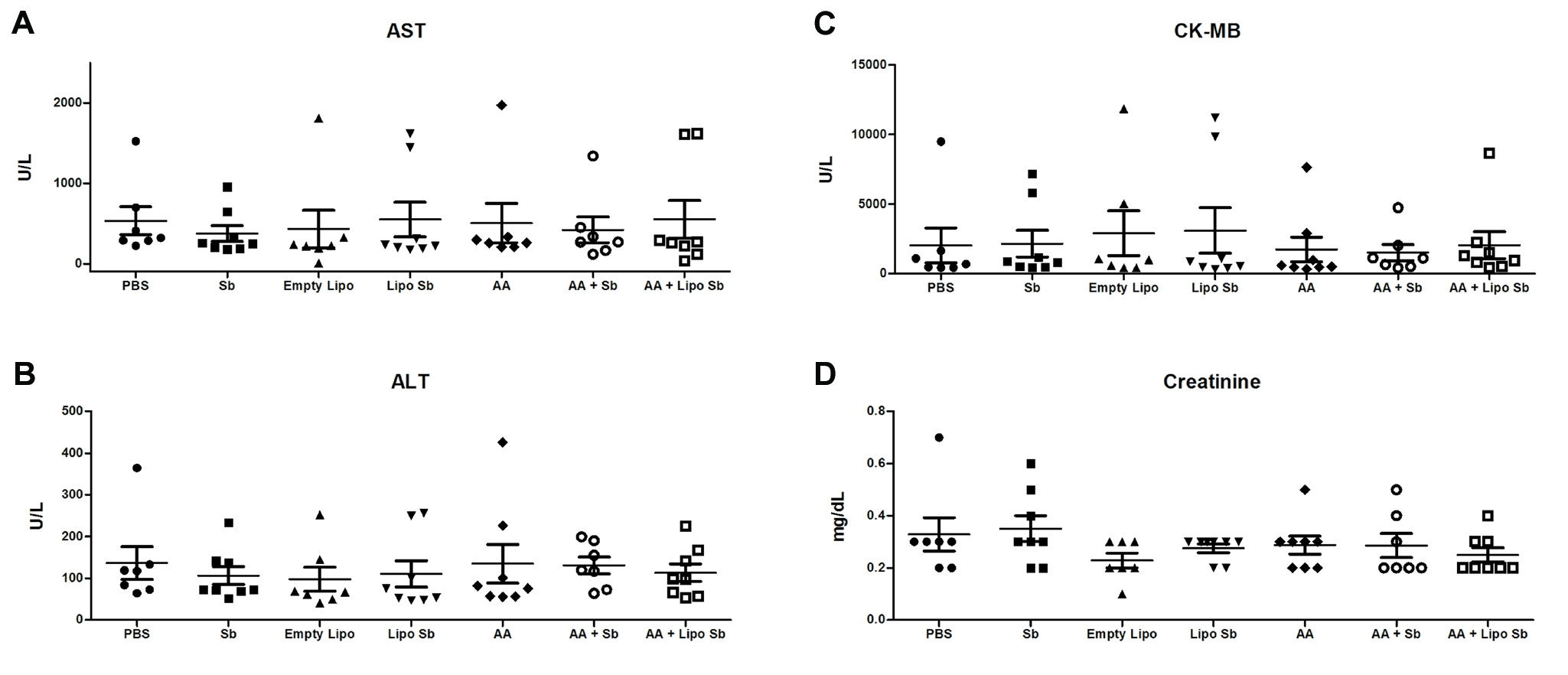

Supplement: Figure S1 — Monitoring liver, kidney and heart functions for L. infantum infected mice submitted to treatment regimens. (PBS) phosphate-buffered saline, (Sb) trivalent antimony, (Empty Lipo) empty liposomes, (Sb Lipo) antimony entrapped liposomes, (AA) ascorbic acid, (AA+Sb) ascorbic acid in association with antimony, (AA+Sb Lipo) ascorbic acid in association with antimony entrapped liposomes. (A) Serum activity of aspartate aminotransferase (AST). (B) Serum activity of alanine aminotransferase (ALT). (C) Serum activity of creatine kinase MB isoenzyme (CK-MB). (D) Serum creatinine concentration in mg/mL. Enzyme activity is expressed as U/L. Results are represented as median ± interquartile range of two independent experiments. No statistically significant differences were observed. (TIF) [file pone.0104055.s001.tif]

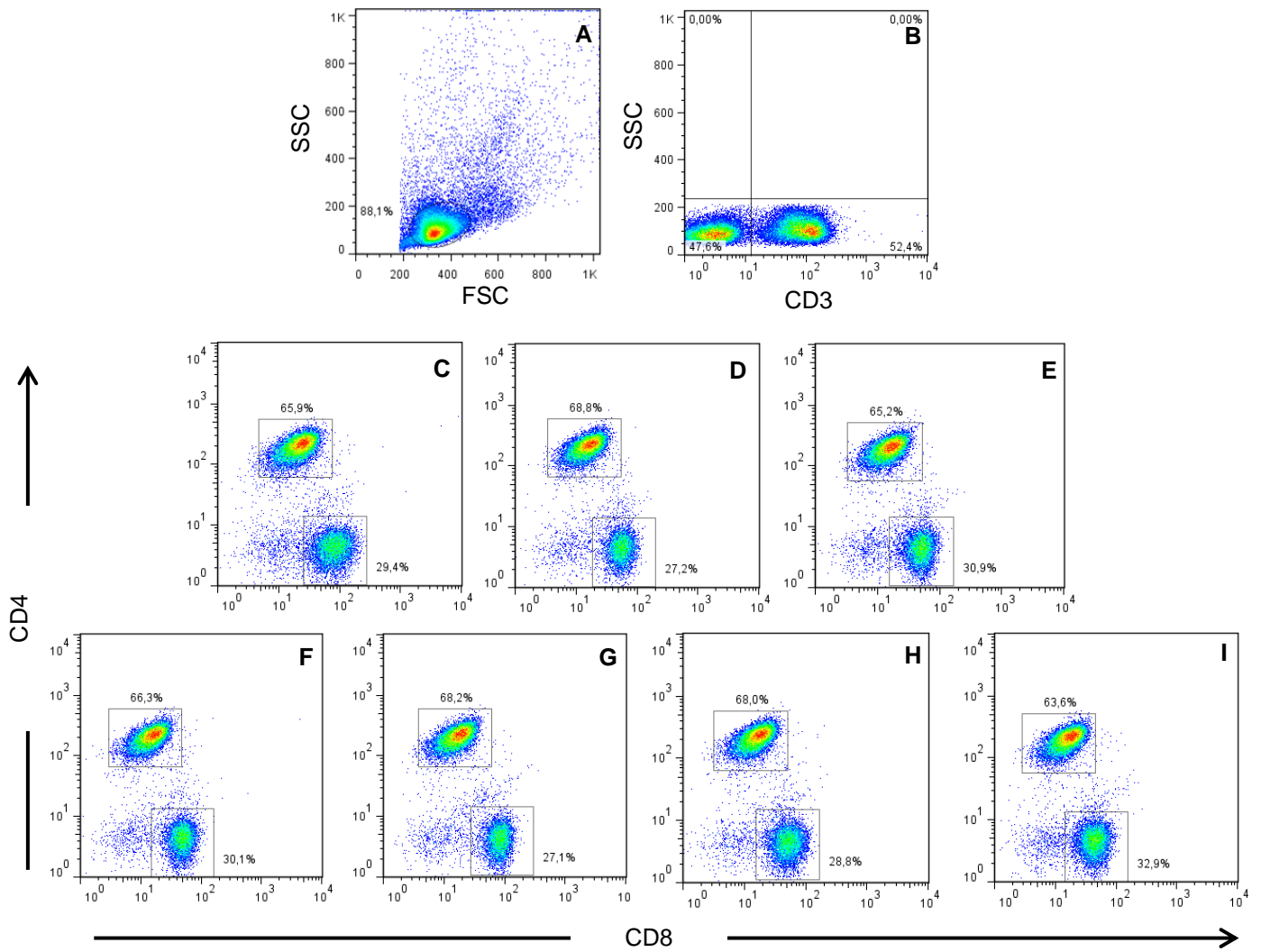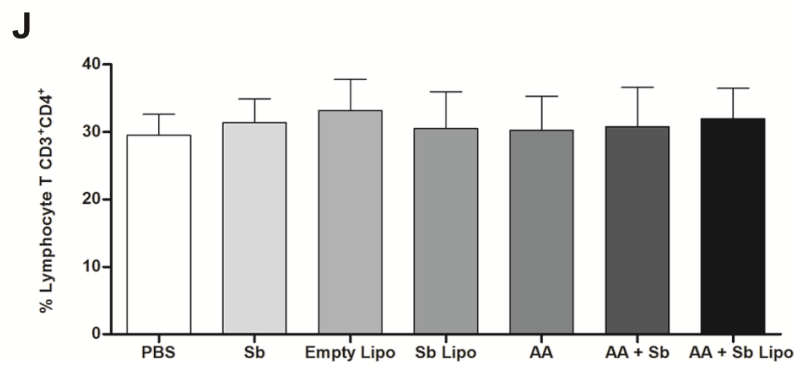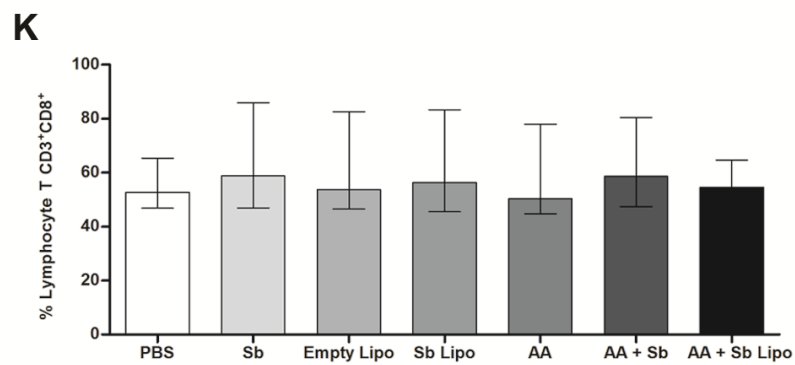

Supplement: Figure S2 — Ex vivo immunophenotyping of T lymphocytes from L. infantum infected mice submitted to treatment regimens. (PBS: A-C) phosphate-buffered, (Sb: D) saline trivalent antimony, (Empty Lipo: E) empty liposomes, (Sb Lipo: F) antimony entrapped liposomes, (AA: G) ascorbic acid, (AA+Sb: H) ascorbic acid in association with antimony, (AA+Sb Lipo: I) ascorbic acid in association with antimony entrapped liposomes. (A) Size and granularity profiles of lymphocytes. (B) Granularity profile of CD3+ cells. (C-I) Profile of T CD8+ and CD4+ lymphocytes. (J) Percentage of T (CD4+) lymphocytes. (K) Percentage of T (CD8+) lymphocytes. In (J) results are represented as mean ± SD whereas for (K) as median ± interquartile range of two independent experiments. No statistically significant differences were observed. (PDF) [file pone.0104055.s002.pdf]

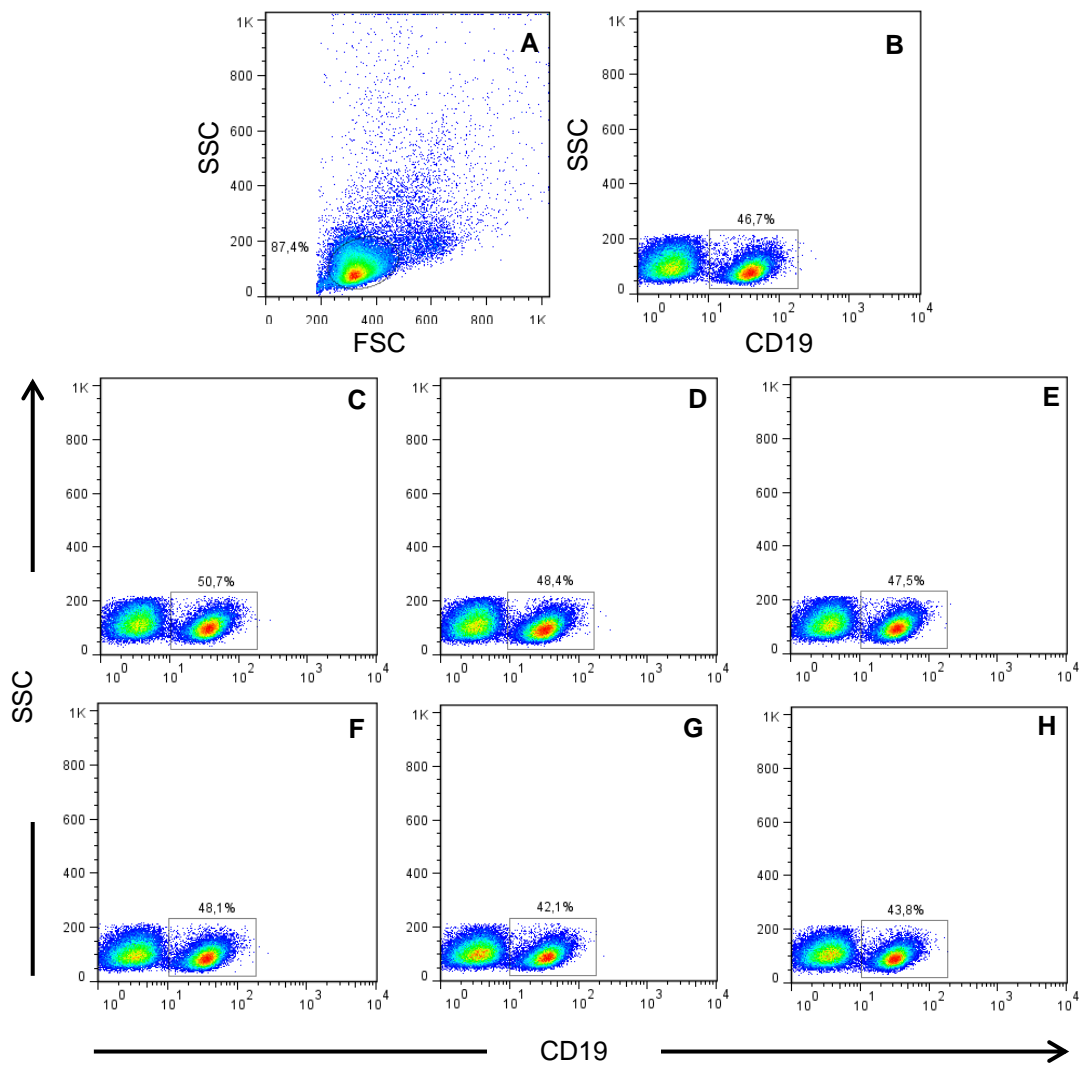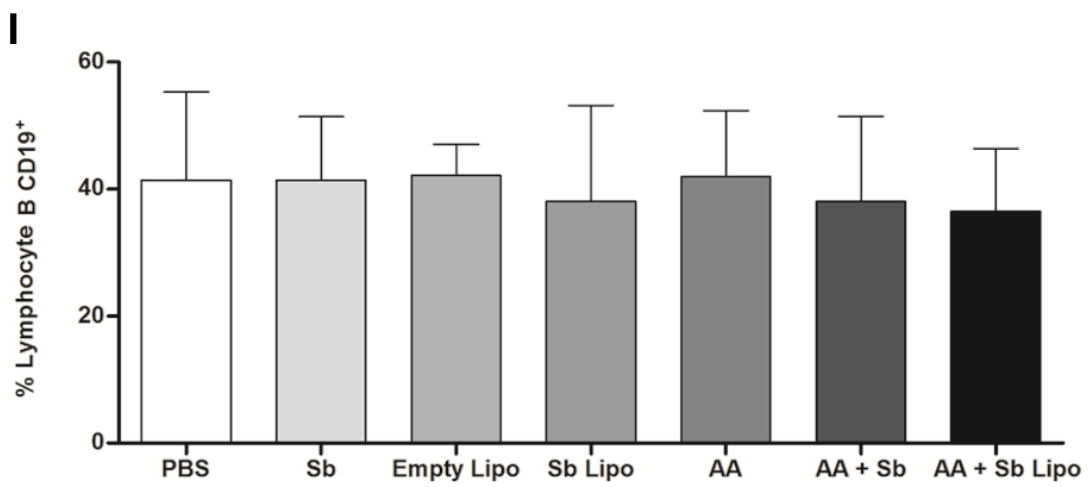

Supplement: Figure S3 — Ex vivo immunophenotyping of B lymphocytes from L. infantum infected mice submitted to treatment regimens. (PBS: A-B) phosphate-buffered, (Sb: C) saline trivalent antimony, (Empty Lipo: D) empty liposomes, (Sb Lipo: E) antimony entrapped liposomes, (AA: F) ascorbic acid, (AA+Sb: G) ascorbic acid in association with antimony, (AA+Sb Lipo: H) ascorbic acid in association with antimony entrapped liposomes. (A) Size and granularity profiles of lymphocytes. (B-H) Granularity profile of CD19+ cells. (I) Percentage of B (CD19+) lymphocytes. In (I) results are represented as mean ± SD of two independent experiments. No statistically significant differences were observed. (PDF) [file pone.0104055.s003.pdf]

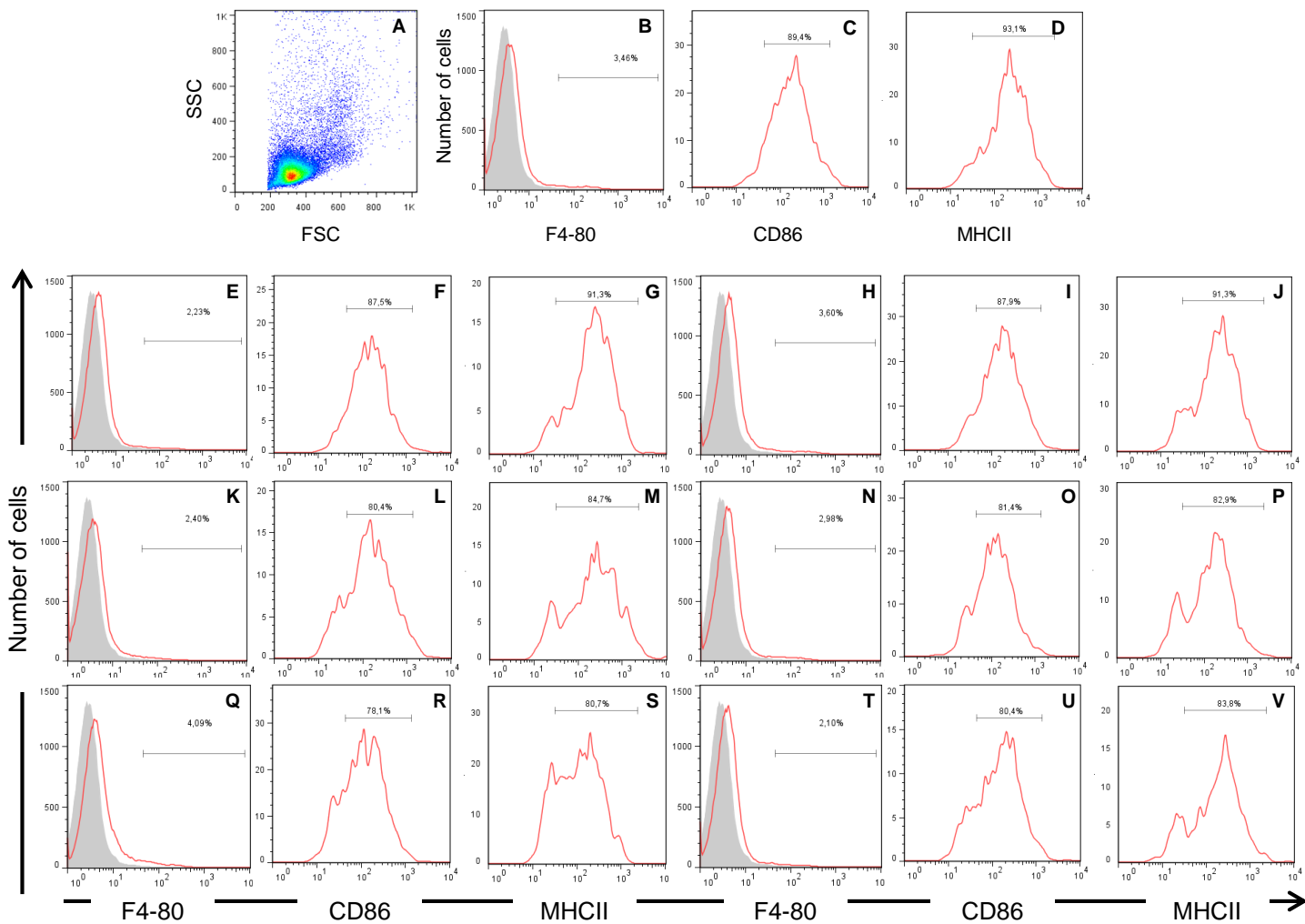

**W**

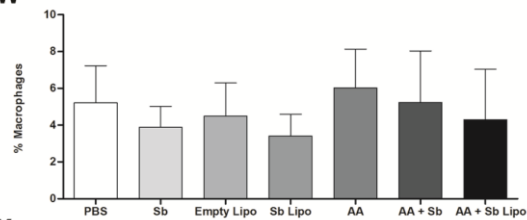

**X**

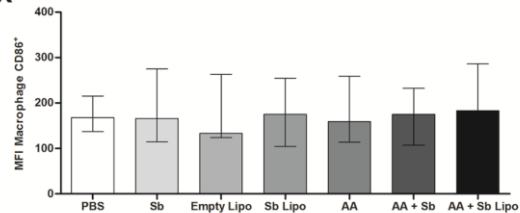

**Y**

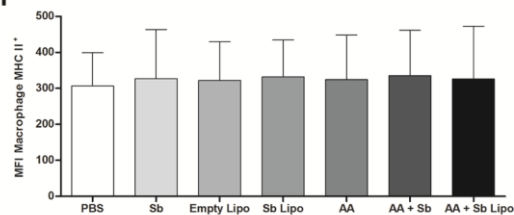

Supplement: Figure S4 — Ex vivo immunophenotyping of macrophages from L. infantum infected mice submitted to treatment regimens. (PBS: A-D) phosphate-buffered, (Sb: E-G) saline trivalent antimony, (Empty Lipo: H-J) empty liposomes, (Sb Lipo: K-M) antimony entrapped liposomes, (AA: N-P) ascorbic acid, (AA+Sb: Q-S) ascorbic acid in association with antimony, (AA+Sb Lipo: T-V) ascorbic acid in association with antimony entrapped liposomes. (A) Size and granularity profiles of splenocytes. (B; E; H; K; N; Q; T) Percentage of F4/80+ cells. (C; F; I; L; O; R; U) Percentage of F4/80+ CD86+ cells. (D; G; J; M; P; S; V) Percentage of F4/80+ MHCII+ cells. (W) Percentage of F4/80 labeled macrophages. (X) Mean fluorescence intensities for F4/80 and CD86 labeled macrophages. (Y) Mean fluorescence intensities for F4/80 and MHCII labeled macrophages. In (W) and (Y) results are represented as mean ± SD whereas for (X) as median ± interquartile range of two independent experiments. No statistically significant differences were observed. (PDF) [file pone.0104055.s004.pdf]
